# A New Optimized GA-RBF Neural Network Algorithm

**DOI:** 10.1155/2014/982045

**Published:** 2014-10-13

**Authors:** Weikuan Jia, Dean Zhao, Tian Shen, Chunyang Su, Chanli Hu, Yuyan Zhao

**Affiliations:** ^1^School of Electrical and Information Engineering, Jiangsu University, Zhenjiang 212013, China; ^2^School of Computer Science and Technology, China University of Mining and Technology, Xuzhou 221008, China; ^3^Changzhou College of Information Technology, Changzhou 213164, China

## Abstract

When confronting the complex problems, radial basis function (RBF) neural network has the advantages of adaptive and self-learning ability, but it is difficult to determine the number of hidden layer neurons, and the weights learning ability from hidden layer to the output layer is low; these deficiencies easily lead to decreasing learning ability and recognition precision. Aiming at this problem, we propose a new optimized RBF neural network algorithm based on genetic algorithm (GA-RBF algorithm), which uses genetic algorithm to optimize the weights and structure of RBF neural network; it chooses new ways of hybrid encoding and optimizing simultaneously. Using the binary encoding encodes the number of the hidden layer's neurons and using real encoding encodes the connection weights. Hidden layer neurons number and connection weights are optimized simultaneously in the new algorithm. However, the connection weights optimization is not complete; we need to use least mean square (LMS) algorithm for further leaning, and finally get a new algorithm model. Using two UCI standard data sets to test the new algorithm, the results show that the new algorithm improves the operating efficiency in dealing with complex problems and also improves the recognition precision, which proves that the new algorithm is valid.

## 1. Introduction

Neural network (NN) is an interdiscipline, and it involves many subjects, such as computer, mathematics, neural, and brain. It is based on the intelligent computation of the computer network imitating biological neural network, which is good at dealing with nonlinear problems and massive calculation. Neural network has the history of more than 70 years and hundreds of neural network models have been proposed, and different network models have their own superiority in dealing with different problems. Radial basis function (RBF) neural network is a three-layer feed-forward network with a single hidden layer; it can approach any continuous function with arbitrary precision, and it has some excellent characteristics, such as structure-adaptive-determination, independent of the initial value of output. Its superiority lies in using linear learning algorithms to complete the work which is done by nonlinear learning algorithms formerly; meanwhile, it maintains the high precision of the nonlinear algorithms; it has the characteristics like best approximation, global optimum, and so on. RBF neural network is widely used [1–3] in the traditional classification problem.

Comparing the RBF neural network with the classic forward neural network such as back-propagation (BP) network [4], the main difference is that BRF neural network has more hidden layer neurons, only one set of layer connection weights from the hidden layer to the output layer; the hidden layer takes the radial basis function as the activation function, generally using Gaussian function [5]; both unsupervised and supervised learning have been used in the training process and so on. In the hidden layer of RBF neural network, each neuron corresponds to a vector of the same length as a single sample, which is the center of neuron. The centers are usually obtained by K-means clustering; this step seems as unsupervised learning; the connection weights from the hidden layer to the output layer are usually obtained by the least mean square (LMS) method, so this step seems as supervised learning. In the RBF neural network, the nonlinear transfer functions (i.e., basis function) do not affect the neural network performance very much; the key is the selection of the center vectors of basis functions (hereinafter referred to as the “center”). If we select improper center, it is difficult for the RBF neural network performance to achieve satisfactory results; for example, if some centers are too close, they will produce approximate linear correlation and then result in lesions on numerical criteria; if some centers are too far, they are short of the requirement of linear processing. Too many centers may easily lead to overfitting, while it is difficult to complete classification tasks if centers are too few [6]. RBF neural network performance depends on the choice of the hidden layer's center, it determines whether the neural network had successful training and can be applied in practice or not.

Genetic algorithm (GA) is developed from natural selection and evolutionary mechanisms; it is a search algorithm with the characters of being highly parallel, randomized, and adaptive. Genetic algorithm uses the group search technology and takes population on behalf of the solution of a group questions. By doing a series of genetic operations like selection, crossover, mutation, and so on to produce the new generation population, and gradually evolve until getting the optimal state with approximate optimal solution, the integration of the genetic algorithm and neural network algorithm had achieved great success and was widespread [7–10]. Using the genetic algorithms to optimize the RBF neural network is mostly single optimizing the connection weights or network structure, [11–13], so in order to get the best effect of RBF, in this paper, the way of evolving both two aspects simultaneously is provided. A new optimized RBF neural network algorithm based on genetic algorithm is established. New algorithm used hybrid coding, that is, taking the binary encoding method to encode the neural network structure and taking the real number encoding method to encode the weights between hidden layer and output layer, so that we can achieve the self-adaptation of adjusting the structure of neural network and the learning of connection weight simultaneously. A good structure has been got; however, the weight optimization is incomplete; it needs to be further optimized. Least mean square (LMS) algorithm [14–16] is chosen, to optimize the connection weights continuously. Finally, a precise RBF neural network has been obtained.

To verity the validity of the new algorithm, this study arranges two experiments, using three UCI standard data sets to test. From the following, some aspects to evaluate the algorithm, such as success training rate, training step, and recognition accuracy rate, are obtained. By comparing with every experiment results, it verifies the superiority of the new optimizing algorithm.

## 2. Genetic Algorithm and RBF Neural Network

### 2.1. The Basic Theory of Genetic Algorithm

Genetic algorithm starts from a population of represented potential solution set; however, the population is composed of a certain number of encoded gene individuals, which is the entities with characteristic chromosome. The main problems of constructing the genetic algorithm are the solvable encoding method and the design of genetic operator. Faced with different optimization methods, we need to use different encoding method and genetic operators of different operation, so they as well as the degree of the understanding of the problems to be solved are the main point determining whether the application of genetic algorithm can succeed.

**Figure 1:**
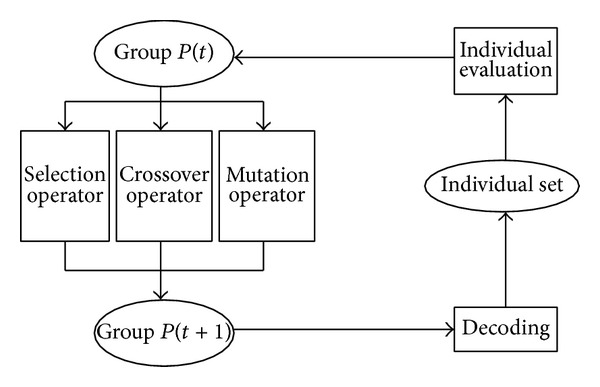
The flow chart of genetic algorithm.

It is an iterative procedure; in each iteration, it retains a candidate solution and sorts them by the quality of the solutions and then chooses some of the solution according some indicators and uses genetic operators to compute it to produce a new generation of candidate solutions. We will repeat this process until it meets some convergence index Figure 1 clearly shows the process of the genetic algorithm.

### 2.2. The Basic Theory of RBF Neural Network

**Figure 2:**
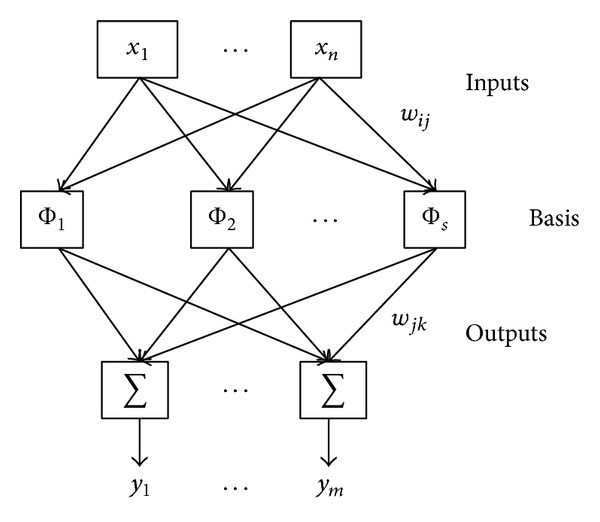
The topology structure of RBF neural network.

The work thought of RBF network is to take RBF as the “basis” of the hidden layer units, so as to construct the hidden layer space. It is a nonlinear function that is symmetrical on the central points and distributed locally, when the central points of the RBF are determined; then the input vector can be directly mapped to the hidden space. But the mapping from the hidden space to the output space is linear, that is, the linear weighting sum of the network unit output; the weight here is the network's adjustable parameters. The RBF network is a three-layer feed-forward network which is composed of input layer, hidden layer, and output layer.  Figure 2 shows the RBF network topology; the hidden layer takes the RBF function as the activation function; generally we use Gaussian function.

Suppose the network has *n* inputs and *m* outputs, the hidden layer has *s* neurons, the connection weight between the input layer and the hidden layer is *w*_*ij*_, and the connection weight between the hidden layer and output layer is *w*_*jk*_.

The training process of RBF network can be divided into two steps; the first step is to learn to identify the weight *w*_*ij*_ without teacher, and the second step is to identify the weight *w*_*jk*_ with teacher. It is a key problem to identify the number of the hidden layer's neurons; usually it starts to train from 0 neurons; the hidden layer neuron is increased automatically by checking the error and repeats this process until the requested precision or the largest number of hidden layer's neurons is achieved.

## 3. Optimized RBF Algorithm Based on Genetic Algorithm

### 3.1. The Thought of GA-RBF Algorithm

Comparing RBF neural network with BP network, RBF can self-adaptively adjust the hidden layer in the training stage according to the specific problems; the allocation of the hidden layer's neurons can be decided by the capacity, the category, and the distribution of the training samples; the center points and its width of the hidden layer's neurons and the hidden layer can be dynamically identified, and it learns fast. Once the architecture of the BP network is identified, the architecture does not change while training; it is difficult to determine the number of hidden layers and its neurons; the rate of convergence of the network is low, and the training has some correlation of the pending sample, the algorithms selection, and the network architecture. It is obvious that the performance of the RBF network is superior to the BP network.

**Figure 3:**
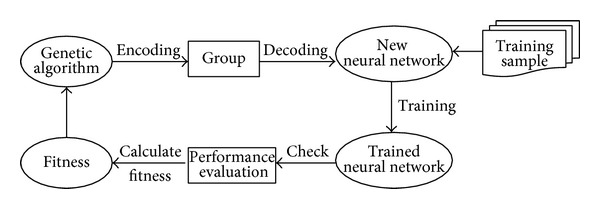
The flow chart of GA-RBF algorithm.

The main content of using genetic algorithm to optimize RBF network includes the chromosome coding, the definition of fitness function, and the construct of genetic operators. The use of GA-RBF optimization algorithm can be seen as an adaptive system; it is to automatically adjust its network structure and connection weights without human intervention and make it possible to combine genetic algorithm with the neural network organically, which is showed as in Figure 3.

#### 3.1.1. Chromosome Encoding

Suppose the number of RBF neural network's maximum hidden neurons is *s* and the number of output neurons is *m*.

Hidden layer's neurons with binary coding, and the coding scheme are as follows:

(1) $$\begin{array}{c} c_{1}c_{2}\cdots c_{s}. \end{array}$$

Here, the number of hidden layer neurons is encoded by binary encoding method, represented by *c*_*i*_, the value of which is 0 or 1. When *c*_*i*_ = 1, it means that the neuron exists; while *c*_*i*_ = 0 it means that the neuron does not exist, and *s* represents the upper limit.

The weights with real encoding, coding scheme are as follows:

(2) $$\begin{array}{c} w_{11}w_{21}\cdots w_{s1}w_{12}w_{22}\cdots w_{s2}\cdots w_{1m}w_{2m}\cdots w_{sm}. \end{array}$$

Here, the weights from hidden layer to output layer was encoded by real number encoding method, and *w*_*ij*_ represents the connection weight from the* i*th output neuron to the* j*th hidden neuron.

The threshold also with real encoding coding scheme is as follows:

(3) $$\begin{array}{c} \theta_{1}\theta_{2}\cdots \theta_{m}. \end{array}$$

Here, the threshold of output layer neuron is also encoded by real number encoding method; *θ*_*j*_ represents the threshold of* j*th output neuron.

So, in conclusion, the complete coding strand of one chromosome is the combination of the structure, connection weight, and threshold, and it is as follows:

(4) c1c2⋯csw11w21⋯ws1w12w22 ⋯ws2⋯w1mw2m⋯wsmθ1θ2⋯θm.

#### 3.1.2. Constructing Genetic Operator

(*1) Selection Operator*. When it comes to the selection operator, in this paper, choose the proportional selection operator and use the roulette wheel selection, which is the most commonly used method in genetic algorithm. The individuals with higher fitness will more likely be selected, while the individuals with lower fitness also have the chance to be selected, so that it keeps the diversity of the population under the condition of “survival of the fittest”.

(*2) Crossover Operator*. We use single-point crossover operator as the crossover operator; each time we choose two individuals of parent generation to crossover so as to generate two new individuals, which are added into the new generation. We will repeat this procedure until the new generation population reaches the maximum size.

We use single-point crossover although the complete procedure uses hybrid encoding; however, the crossover operation for binary encoding and real encoding is the same. The strategy of elitism selection is used here, that is, to retain several individuals with highest fitness to the next generation directly; this strategy prevents the loss of the optimal individual during the evolution.

(*3) Mutation Operator.* Mutation operator uses reversal operator, as it uses hybrid encoding; different operations are applied to different code system. Binary encoding uses bit-flipping mutation; that is to say, some bit of the chromosome may turn from 1 to 0 or 0 to 1. For real encoding, we use Gaussian mutation; that means some gene of the chromosome will add a random Gaussian number.

#### 3.1.3. Calculate Fitness

Fitness function evaluation is the basis of genetic selection, so it will directly affect the performance of genetic algorithm. Therefore, the selection of fitness function is very crucial; it directly affects the speed of genetic algorithm convergence and whether we can find the optimal solution.

The original data sets are divided into training data sets and testing data sets, using the network training error and the number of hidden neurons to determine the RBF neural networks' corresponding fitness of the chromosomes. Suppose the training error is *E*, the number of hidden layer neurons is *s*, and upper limit of the number of hidden layer neurons is *s*_max⁡_. So the fitness *F* is defined by

(5) $$\begin{array}{c} F=C-E\times \frac{s}{s_{\max}}. \end{array}$$

In the formula, *C* is a constant number; this formula ensures that the smaller the network size (fewer hidden layer neurons) and the smaller the training error, the higher the corresponding fitness of chromosome.

#### 3.1.4. Parameters of RBF Neural Network

In the classical RBF neural network, there are three parameters that can be adjusted: centers and its width of the hidden layer's basis function and the connection weights between hidden layer and output layer. Construction of the classical RBF neural network generally adopts the following rules.

(*1) Basis Function Centers*. By selecting basis function centers according to experience, if the distribution of training sample can represent the problem, in other words, we can select the *s* centers according to the experience; the spacing is *d*; the width of the selected Gaussian function is

(6) $$\begin{array}{c} \sigma =\frac{d}{\sqrt{2s}}. \end{array}$$

(*2) Basis Function*. We use K-mean cluster method to select the basis function; the center of each cluster is regarded as the center of basis functions. As the output is linear unit, its weights can be calculated directly by LMS method.

We use iterative formula (7) to modify the training error, so we can get the following optimal neural network algorithm:

(7) $$\begin{array}{c} e=\overset{n}{\underset{k=1}{\sum }}{\left(t_{k}-y_{k}\right)}^{2}. \end{array}$$

Here, *e* is the error faction, *t*_*k*_ is the actual value, and *y*_*k*_ is the output of neural network.

### 3.2. The Basis Steps of GA-RBF Algorithm

The GA-RBF neural network algorithm basis step is descried as follows.

**Step 1 .**

Set the RBF neural network, according to the maximum number of neurons in the hidden layers; use K-clustering algorithm to obtain the center of basis function; use formula (6) to calculate the width of the center.

**Step 2 .**

Set the parameters of the GA, the population size, the crossover rate, mutation rate, selection mechanism, crossover operator and mutation operator, the objective function error, and the maximum number of iterations.

**Step 3 .**

Initialize populations *P* randomly; its size is *N* (the number of RBF neural network is *N*); the corresponding network to each individual is encoded by formula (4).

**Step 4 .**

Use the training sample to train the initial constructed RBF neural network, whose amount is *N*; use formula (7) to calculate the network's output error *E*.

**Step 5 .**

According to the training error *E* and the number of hidden layer neurons *s*, use formula (5) to calculate the corresponding chromosome fitness to each network.

**Step 6 .**

According the fitness value, sort the chromosome; select the best fitness of the population, denoted by *F*_*b*_; verify *E* < *E*_min⁡_ or *G* ≥ *G*_max⁡_; if yes, turn to Step 9; otherwise turn to Step 7.

**Step 7 .**

Select several best individuals to be reserved to the next generation New*P* directly.

**Step 8 .**

Select a pair of chromosomes for single-point crossover, to generate two new individuals as members of next generation; repeat this procedure, until the new generation reaches the maximum size of population *Ps*; at this time, the coding will be done separately.

**Step 9 .**

Mutate the population of new generation; binary coding part and real number coding part should use different mutation strategies. Then the new population is generated; set *P* = New*P*, *G* = *G* + 1; return to Step 4.

**Step 10 .**

Get the optimal neural network structure, and the iteration of genetic algorithm is terminated, which means the optimizing stopped.

**Step 11 .**

The new neural network's weight learning is not sufficient, so use LMS method to further learn the weights. End of the algorithm.

The significance of establishing new model is that to optimize neural network structure, to determine the number of hidden layer neurons and the center of the basis function, to optimize the connection weight and threshold, in order to improve the training speed and convergence, to save network running time, and then to improve the operating efficiency of network and the ability of dealing with problems.

## 4. Experiment

In order to verify the validity of the new algorithm, we use several algorithms for comparison. And mark every algorithm as follows.

1. The classical RBF algorithm, with least mean square (LMS) method to solve the weights from the hidden layer to output layer, is denoted by RBF.
2. Use GA to optimize the network structure and weights of the RBF algorithm simultaneously; denote GA-RBF.
3. Then use LMS method for weights further learning; get the algorithm; denote GA-RBF-L.

Use training sample to train each algorithm and test by simulation sample. And then get six measurement indexes: training success rate, training error, test error, classification accuracy rate, number of hidden neurons, and operation time, so that we can measure the merits of the algorithm.

### 4.1. Test Preparation

By using LMS method to further learn the weights, the maximum number of iterations is 3,000, the learning rate is 0.1; the maximum size of the neural network is 90.

The maximum number of GA iterations is 600, the population size is 50, the crossover rate is 0.9, and the mutation rate is 0.01. We use the C++ and Matlab for hybrid programming.

In order to better illustrate the validity of new algorithm, we use two UCI data sets for testing; one data set is waveform database generator (V2) [17], and the other data is wine data set [18].

The experiments are run on Intel Core2 Duo CPU E7300 2.66 GHz, RAM 1.99 GB.

### 4.2. Test 1

The waveform database generator (V2) data set has 5000 samples, and each sample has 40 features, which is used in waveform classification. In this paper, we select the front 600 samples to test, among 500 as training samples, the remaining 100 as the simulation samples. Every algorithm repeats the test 50 times and then records the best ones' result. The results of each algorithm are listed in Table 1.

### 4.3. Test 2

In order to further verify the validity of new algorithm, we use another UCI standard data set to test and also verify the generalization ability. The wine data set has 178 samples, 13 features, and 3 classes.

Select the front 170 samples to test, randomly dividing the wine data set into training samples and simulation samples by the ratio 4 : 1. Every algorithm runs 50 times, each test is random and then records the average value, listing them in Table 2.

### 4.4. Results

Tables 1 and 2 illustrate that, from the training success rate (the success times within 50 training times) aspect, GA optimized RBF algorithm is superior to the traditional RBF algorithm; from the training error and test error aspect, RBF and GA-RBF-L algorithm are equivalent, or slightly better than GA-RBF algorithm; from the operation time aspect, the operation time of GA optimized RBF algorithm is slightly longer, because running the genetic algorithm will take longer time; from the recognition precision aspect, the GA-RBF-L algorithm's classification precision is the best.

## 5. Conclusion and Discussion

In this paper, we propose a new algorithm that uses GA to optimize the RBF neural network structure (hidden layer neurons) and connect weight simultaneously and then use LMS method to adjust the network further. The new algorithm optimized the number of the hidden neurons and at the same time completely optimized the connection weights. New algorithm takes longer running time in genetic algorithm optimizing, but it can reduce the time which is spent in constructing the network. Through these two experiments analysis, the results show that the new algorithm greatly improves in generalization capability, operational efficiency, and classification precision of RBF neural network.

The network structure will affect the generalization capability of the algorithm, comparing RBF, GA-RBF, and GA-RBF-L; while the RBF algorithm gets the small training error, its recognition precision is not as good as GA-RBF-L algorithm whose hidden layer neurons are fewer. Genetic algorithm is effective for the evolution of the network structure; it can find a better network structure, but it is not good at optimizing connection weights. After 500 generations of iteration, the downtrend of the training error turns slow, so that we use LMS method further to adjust the weights and then get the optimal algorithm. The new algorithm is a self-adapted and intelligent algorithm, a precise model; it is worthy of further promotion.

## Figures and Tables

**Table 1:** The comparison of the performance of each algorithm for waveform database.

Neural networks algorithm	Traditional RBF	GA-RBF	GA-RBF-L
Training success rate, %	86	100	100
Training error	0.22	0.36	0.29
Test error	1.78	1.97	1.61
Number of hidden neurons	44	28	28
Operation time, s	1.21	1.62	1.62 + 0.22
Classification accuracy, %	89	87	97

**Table 2:** The comparison of the performance of each algorithm for wine data set.

Neural networks algorithm	Traditional RBF	GA-RBF	GA-RBF-L
Training success rate, %	90	100	100
Training error	0.37	0.53	0.32
Test error	0.89	1.06	0.67
Number of hidden neurons	14	8	8
Operation time, s	0.27	1.53	1.53 + 0.19
Classification accuracy, %	90.94	86.88	96.35
